# Sports Activities in Children with Cerebral Palsy: A Narrative Review

**DOI:** 10.3390/medicina60030457

**Published:** 2024-03-09

**Authors:** Domenico M. Romeo, Giulia D’Amario, Giulia Brunozzi, Valentina Napoli, Marianna Villa, Chiara Arpaia, Chiara Velli, Francesca Sini, Claudia Brogna

**Affiliations:** 1 Pediatric Neurology Unit, Fondazione Policlinico Universitario A. Gemelli, IRCCS, 00168 Rome, Italy; giuliadamario@gmail.com (G.D.); valenap86@gmail.com (V.N.); marianna.ivilla@gmail.com (M.V.); arpaia.chiara@gmail.com (C.A.); chiara.velli@unicatt.it (C.V.); franc.sini@hotmail.it (F.S.); claudiabrogna@yahoo.it (C.B.); 2 Pediatric Neurology Unit, Università Cattolica del Sacro Cuore, 00168 Rome, Italy; giuliabrunozzi94@gmail.com

**Keywords:** cerebral palsy, sport, children, rehabilitation

## Abstract

Physical exercise is known to have beneficial effects on psychosocial well-being and cognitive performance. Children with cerebral palsy (CP) showed lower levels of physical activity (PA) than healthy children; this fact, in addition to the basic clinical condition, increased the sedentary habit with a psychological impact and motor impairment of these children. Furthermore, children and adolescents with CP are less committed to sports activities than typically developing children of the same age. The aim of the present narrative review was to increase the amount of knowledge regarding the effectiveness and importance of specific and individualized sports in children with CP. A comprehensive search of MED-LINE and EMBASE databases was performed, including specific search terms such as “cerebral palsy” combined with “sport”, “physical activity”, and the names of different sports. No publication date limits were set. We included studies with an age range of 0–18 years. The main results pointed out that most of the sports improved motor function, quality of life, and coordination in children and adolescents with CP. Physicians, therapists, and parents should become aware of the benefits of sports activities for this population of patients. Specific sports activities could be included as a usual indication in clinical practice in addition to rehabilitation treatment.

## 1. Introduction

Adequate daily physical activity (PA) is associated with important health benefits in all children (8), including children with disabilities [1]. The American College of Sports Medicine published guidelines about the importance of sports and PA in healthy populations in order to improve functional capacity, decrease the incidence of obesity and type 2 diabetes, and reduce the development of osteoporosis, fracture incidence, and the risk of cardiovascular complications [2].

The health benefits are also psychological and include improvement in symptoms of depression, anxiety, and self-conception. For this reason, the World Health Organization recommends at least 60 min of moderate to vigorous physical activity per day, including muscle and bone strengthening activities at least three days per week [1]. However, children with cerebral palsy (CP) showed lower levels of PA and less involvement in sports activities than healthy children; therefore, it was common to have an increase in sedentary behavior and a resulting motor impairment. Cerebral palsy (CP) is a group of permanent, but not immutable, disorders of movement and posture of motor function due to a non-progressive lesion or abnormality of the development of the immature brain [3,4,5]. Children with CP experience varying degrees of muscle weakness, muscle contracture, spasticity, impaired balance, and coordination that limit their functional capacity to perform activities such as running, jumping, climbing, and cycling [4,5]. These difficulties reduce the capacity to perform typical childhood actions and contribute to low habitual physical activity that results in energy expenditure [6,7]. 

There are numerous articles in the literature evaluating PA in children with cerebral palsy, although the majority of these studies examine the use of PA in a rehabilitative setting and not strictly in a recreational sports context [8,9,10,11,12,13,14,15,16,17,18,19]. The effectiveness of physiotherapy and neurodevelopmental rehabilitation treatment in children with CP is well known [8]. On the other hand, less is known about the potential benefit of sports. Sports activities could provide chances for children with CP to develop independence for social integration, participation, quality of life, and PA; furthermore, it is very important for children with motor disabilities to have realistic opportunities to develop motor function through participation in sports [18]. Joining in sports activities has reported increased engagement, motivation, and participation compared to individual interventions. Additionally, participation in sports activities has been considered an alternative to prolonged physiotherapy intervention in adolescence and into adulthood [18]. 

However, according to a recent systematic review, children with CP of school age are 30% less committed to sports than typically developing children of the same age [19].

The aim of the present research is to review data published in the literature about the effectiveness and importance of specific and individualized sports in children with CP in order to evaluate the possibility of including sports activity as a usual indication in clinical practice in addition to rehabilitation treatment.

## 2. Methods

### 2.1. Search Criteria 

A comprehensive search of the following electronic databases was performed: MEDLINE and EMBASE. Search terms used were “cerebral palsy”, which was combined with “sport”, “physical activity”, and the names of different sports (“dance”, “swimming”, “tennis”, “martial arts”, “basketball”, “soccer”, “baseball”, and “adaptive cycling”). Duplicates were excluded prior to the retrieval of references. Abstracts for each reference were obtained and screened using the following criteria: 

### 2.2. Inclusion Criteria

Studies were eligible for inclusion if they were written in English and were human-based. All the studies were first selected, looking for the presence of a clinical association between “cerebral palsy”, “pediatric cerebral palsy” or “CP”, and “Sport”, “physical activity”, “nintendo”, and the names of individual sports (“dance”, “swimming”, “tennis”, “martial arts”, “basketball”, “soccer”, “baseball”, and “adaptive cycling”) and reporting the details of interventions. No publication date limits were set. We included studies with an age range of 0–18 years. 

### 2.3. Exclusion Criteria 

Studies were excluded if they analyzed sports activities performed within rehabilitation activity. This was to examine the use of PA in a recreational sports context and not as an individual intervention.

### 2.4. Data Extraction and Analysis 

The title and abstracts of the studies were independently examined for suitability by two authors (GD, GB) and critically checked by a third independent reviewer (D.M.R.); conflicting viewpoints were discussed until a consensus was reached. 

A total of 46 studies were initially identified; after a review of the full text, 31 were excluded, as they included neurodegenerative disorders or physical activity within rehabilitation activity or as they were reviewed. The remaining 15 articles, comprising a total of 401 children and 8 types of sports and video games, were included in the present review (details are reported in Table 1). To improve the quality of the manuscript, the SANRA (Scale for the Assessment of Narrative Review Articles) was also performed, with a sum score of 12 (maximal score possible).

## 3. Results

### 3.1. Dance Activity

Dance was explored in four papers. Hee Joung He et al. reported on 13 children with CP who completed 12 weeks of a creative dance program offered in 2-hour sessions, twice per week, for a total of 24 sessions [20]. The results showed a significant improvement in movement and gait capacity; more specifically, gross motor function measure (GMFM)-88 dimensions D and E, walking speed, cadence, step and stride lengths, and sagittal ranges of motions of the hip and ankle ROM during walking improved significantly at the end of the sessions. A significant decrease in the time of opposite foot off and first double-limb support time was also observed, whereas the percentage of single-limb support time increased. Citlali Lopez-Ortiza et al. [21] examined the perception of children with CP (n. 16), parents (n. 16), and therapists (n. 13) on the therapeutic benefit of the dance program. The classes were held once weekly in sessions of five to eight weeks, depending on location availability. Children reported high enjoyment levels and a desire for more classes; the parents reported a perceived therapeutic benefit, and the therapists viewed the class as a positive adjunct to therapy. In another study, the same authors [22] evaluated the effects of a targeted dance class (TDC) in twelve children with cerebral palsy. The Pediatric Balance Scale (PBS) showed statistically significant improvements in the TDC intervention group immediately after the intervention and at 1-month follow-up but not in the control group. Claire Cherriere et al. [23] evaluated the effects of a dance intervention on balance and the effects of this intervention on walking speed, attention, and rhythm production in adolescents with CP. A total of 10 children with spastic CP were subjected to dance classes of 60 min twice a week for 10 weeks. The results showed a significant balance improvement with a significant improvement in the PBS total score. 

### 3.2. Swimming

Swimming was explored in three papers. Declerk et al. [24] reported on fourteen youths with CP (aged 7 to 17 years) during a 20-week follow-up period (2/week, 40–50 min). Levels of enjoyment were high. Walking (walking distance at maximum walking speed) and swimming skills (balance control and movement in water) improved significantly more in the swimming than in the control group, whereas fatigue and pain did not increase. All youth in the swimming intervention group had high levels of enjoyment. 

### 3.3. Swimming and Other Sports 

Two articles compared swimming and other sports. Luzanira Correia Feitosa et al. [25] aimed to evaluate the effect of adapted sports on 17 children/adolescents, age range 6–18 years with spastic CP. The treatment consisted of swimming and a side soccer program for one year. Results showed benefits in quality of life in the dimensions of transfer and mobility, upper extremity function, and global function. In addition, the authors found a significant improvement in the biopsychosocial profile, considering attention disorder syndrome and attention hyperactivity deficit disorder assessed using the CBCL (Child Behavior Checklist for ages 6–18). Children/adolescents with diplegia obtained greater benefits than those with hemiplegia in relation to comfort and pain (*p* = 0.02) and global dimension. Instead, Sandy A. Ross and collaborators [26] determined the effect of participation in sports programs on walking ability and endurance over time in 97 children with CP; they attended a summer sports program, including swimming and the following activities twice a week: tennis, dance, martial arts, basketball, soccer, baseball, and adaptive cycling. The results showed significant improvements in the Timed Up and Go (fall risk and measurement of the progress of balance, sit-to-stand, and walking), modified 6-minute walk distance, and 25-foot walk/run over time.

### 3.4. Golf

Golf training was explored in one article only. Gercek et al. [27] investigated 19 children with unilateral cerebral palsy, divided into two groups; each of them received either virtual or traditional golf training for over 12 weeks with three days of a 60-min session/day. Both training methods were associated with improved lower extremity flexibility and muscle strength, aerobic endurance, and GMFM-88 compared with the pre-training baseline values. Virtual golf has an important advantage over traditional golf as it is less expensive and more accessible for people with disabilities. 

### 3.5. Informatic Systems (Computers/Video Games) and Other Sports

The use of informatic systems (computers/video games) to support sports activities and the possible effect on patients with cerebral palsy were explored in seven papers [27,28,29,30,31,32,33]. Tony Szturm et al. [28] provided the therapeutic value of a novel computer game-based dual-task balance exercise program. Ten children with CP received a game-based dual-task (DT) balance in which children were required to stand and balance on an unstable surface while playing various computer games for 12 weeks at a frequency of three sessions per week. The results showed that the experimental group reached greater improvements in the Pediatric Balance Scale, GMFM-88, and balance measures than the 10 children subjected to conventional balance training. Hsieh-Chun Hsieh et al. [29] analyzed the use of a gaming platform requiring multidimensional trunk movement. The intervention group (*n* = 20) had to repeatedly play video games with the gaming platform, and the control group (*n* = 20) played the same games using a computer mouse for a 50-minute session for 12 weeks. Participants in the intervention group had better balance performance compared with the control group, and, in particular, there was a significant difference in center-of-pressure sway excursion and different measures of balance over time. In another study, Hsieh-Chun Hsieh et al. [30] examined a total of 56 children with cerebral palsy and randomly divided them into two groups: experimental and control. The children in the experimental group underwent 12 weeks of activities using their feet to play computer games with the proposed balance board, whereas those in the control group played personal computer games with a computer mouse in the standing position. In the analysis of covariance, the proposed new balance board used for personal computer games decreased postural sway and improved the performance of the functional balance tests. 

Devrim Tarakci et al. [31] studied the effects of Nintendo WII fit video games on balance in children with mild cerebral palsy (GMFCS I–II–III). The treatment schedule consisted of 2 days per week (50 min per session) for a total of 12 weeks. The treatment was divided into two groups. Both groups attended a neurodevelopmental treatment (NDT); a conventional balance training program (20 min) was applied in the control group, and a Wii-Fit balance-based video game program was used in the Wii group (20 min). The Wii group presented a significant improvement in all the outcomes evaluated (Functional Forward Reach Test, Functional Sideways Reach Test, Timed Get Up and Go Test, Sit-To-Stand Test, Nintendo Wii-Fit^®^ Balance and Game Scores, 10-meter walking test, 10 steps climbing test, The Functional Independence Measure for Children). Valeska Gatica-roJas et al. [32] compared the effects of Wii therapy and standard physiotherapy on the performance of standing balance in children and adolescents with cerebral palsy to determine the post-treatment effectiveness of Wii therapy and standard physiotherapy. Patients with spastic hemiplegia and spastic diplegia aged 7 to 14 years and level I or II of GMFCS were selected and randomly divided in Wii therapy or standard physiotherapy. In each group, patients received three sessions per week over a period of 6 weeks. Standing balance was assessed at baseline and every 2 weeks. Results showed that Wii therapy was better than standard physiotherapy in improving standing balance in patients with spastic hemiplegia only, but effectiveness waned 2–4 weeks after the end of the intervention. Furthermore, Ji-Hye Do [33] examined the impact of virtual reality-based bilateral arm training on the motor skills of children with hemiplegic cerebral palsy. A Nintendo Wii game was played for 30 min in each of the 12 sessions. The results showed an improvement in upper limb motor skills on the affected sides and in bilateral coordination ability after virtual reality-based bilateral arm training for all subjects. Oleh Kachmara et al. [34] performed a study in order to assess the changes in balance function in children with cerebral palsy (CP) after two weeks of daily training with personalized balance games. The experimental group used personalized balance games available from the Gamification for Better LifE (GABLE) online serious gaming platform. Children from the control group played Nintendo Wii games using a handheld Wii remote. Both groups received the same background treatment. After a training of two weeks, the Trunk Control Measurement Scale (TCMS) and Dynamic Balance Test (DBT) increased significantly in the experimental group only. 

## 4. Discussion

Children with CP present a low frequency of PA during daily activities, although sustaining a physically active lifestyle is essential for youths and adults with CP to achieve and maintain functional capability [35]. In the past, PA was not considered to be appropriate for this group of children, probably due to increased spasticity and muscular stiffness, leading instead to an overall reduction in mobility. However, several studies have found the effectiveness of PA on spasticity [11] and mainly in improving isometric muscle strength and improving mobility, suggesting an improvement when a rehabilitation exercise was targeted to a specific muscular group [12]. An ankle and knee strength increase can have an impact on gait kinematics, motor function, and quality of life. Moreover, no adverse effect on muscle spasticity could be reported when the training program was less than 12 weeks, as the initial increases in strength are a result of neural changes and not muscle hypertrophy.

Moreover, a systematic review demonstrated that strengthening interventions produced significant improvements in strength and physical performance for individuals with CP [19]. It is also important to find PA that should be fun, with no increase in pain during exercise and with no increased risk for injury in youth with CP [36].

In the present narrative review, we analyzed the possible benefits that children can gain from such PA as sports, not as a rehabilitative activity. The main results point out for the first time that most of the sports performed may improve functional performance, quality of life, and coordination in children and adolescents with CP. 

All sports included in the present review established a global improvement in motor function, especially in upper limb mobility due to the use of video games and upper–lower limbs due to sports activities, with no clear collateral effects. Some sports activities reported specific motor function improvements. Playing with Nintendo Wii [31,32,33] resulted in an improvement in upper limb motor skills on the affected sides and in bilateral coordination abilities after virtual reality-based bilateral arm training. Also observed were changes in bilateral hand coordination abilities through the virtual game of basketball (in particular, putting the ball through the hoop). Therefore, bilateral upper limb exercises may improve not only the affected upper limb motor skills but also bilateral hand coordination and the quality of movement during daily living activities. 

Walking abilities improved after dance practice, swimming, and participation in sports programs, including different activities like tennis, dance, martial arts, basketball, soccer, baseball, and adaptive cycling. The results showed a significant improvement after dance practice [20,21,22,23] in GMFM-88 dimensions D and E, walking speed, cadence, step, stride length, and sagittal ranges of motions of the hip and ankle ROM during walking. Mainly, the findings support the utility of the GMFM-88 dimensions E score as a predictor of locomotor performance and emphasize the positive impact of creative dance on gross motor function and gait performance in adolescents with CP. Moreover, these sports activities reported a significant decrease in the time of opposite foot off and first double-limb support time, with an increase in the percentage of single-limb support time. Creative dance has the goal for the participants to develop their own movements and to explore ways of incorporating movement through the learning of movement concepts. The improvements in the sagittal plane hip and ankle ROM due to this activity may explain the improvement in spatiotemporal gait parameters, similarly reported in adults with chronic stroke [37]. Improvement in hip extension and flexion ROM also contributes to increased stride length and walking speed [20].

Furthermore, dance practice and the use of video games improved static and dynamic balance. The use of a gaming balance board [28,29,30,31,32,33,34] for the personal computer decreased the postural sway and improved the performance of the functional balance tests, especially in center-of-pressure sway excursion (CoP sway), Berg Balance scale (BBS), and TUG (Timed Up and Go) assessments. These data are of great interest in daily practice as poor balance, typical of children with CP, diminishes posture adjustment during the performance of functional tasks of daily living, such as walking and toileting. These findings demonstrated that weight shift practice with computer feedback influences standing balance in children with CP [38]. 

The use of video games and the practice of sports also led to an improvement in the biopsychosocial profile; mainly, swimming led to a reduction in hyperactive–inattentive symptomatology [25], and dance showed benefits in terms of better participation in the activities to be carried out and in the awareness of their physical perception [21]. These benefits have been noted not only by the patients themselves and caregivers but also by the therapists, confirming that recreational activity can be a positive implementation with standard rehabilitation therapy. 

The analyzed studies have not shown specific collateral effects due to the practice of sports or video games. The referral of children/adolescents with CP is still limited in our field; the functional limitations could have been one of the reasons. 

Nonspecific differences were observed considering the different types of CP in most of the studies. Luzanira Correia Feitosa et al. [25] reported more benefits in children with diplegia than those with hemiplegia concerning pain and comfort after swimming and soccer activities; mainly, children with hemiplegia presented more functionality, less difficulty in balance, and so a more favorable motor prognosis, which could explain a greater benefit for the diplegic participants. This is also explainable because, in hemiplegia, the sensorimotor system of one of the sides of children remains relatively intact. On the contrary, Gatica-Rojas et al. [32] found that children with hemiplegia only benefited from Wii therapy on posturographic measures of balance. Probably, children with hemiplegia are better able to use proprioceptive and somatosensory information deriving from the unaffected limb, which may have been enhanced by the Wii therapy. 

In considering the validity of our conclusions, the potential effects of some methodological limitations should be considered, as they may have affected the analysis of the reviewed papers. As the present review was not structured as a meta-analysis, no specific and statistical combination of the results from all the studies reviewed was performed.

Furthermore, the variability in the cohorts, in terms of the research design and the characteristics of the population studied (such as the wide age range) and the small number of samples (10 studies analyzed a sample of less than 30 subjects, and the maximum sample considered was 97 patients), made pooling data unrealizable and prevented the drawing of conclusions that can be generalized to the general population. Finally, some studies did not report a control group or gender differences, and most of them included patients with GMFCS I–III; only two studies included patients with GMFCS IV. 

Even with these limitations, the analysis of the studies included helped to confirm the importance of recreational physical activities for a global improvement in motor function as well as of psychosocial aspects, resulting in a general improvement in the quality of life of children with CP. Participating in sports is an important part of childhood development; however, accessing appropriate activities and information is a challenge for children with motor disabilities due to numerous barriers, including children’s motor impairment and weakness, accessibility and facilities in sports programs, and acceptance of the child’s disability [39]. There are many adaptive sports opportunities ranging from the recreational level to the competitive one; some sports, such as football 7-a-side, boccia, and Frame Running (a bike with a three-wheeled frame where the athlete is supported by a saddle and body plate) were designed for athletes with motor disabilities like CP; whereas others, such as Paralympic swimming, cover a wide array of physical disabilities. A recent systematic review also identified Modified Sports as a promising intervention requiring further high-level research [40].

## 5. Conclusions

Physicians, therapists, and parents should become aware of the benefits of sports activities for children and adolescents with CP, such as the availability of modified sports for children and adolescents with CP. 

We suggest including, in addition to the rehabilitation process, specific sports activities that patients may carry out in groups of healthy peers, working on aspects of social skills. In addition, the possibility of having technological devices could lead to greater compliance on the side of the patient and, consequently, to the regular performance of support recreational activities to be carried out in the domestic environment. Finally, it is highly recommended to assess the perceived level of enjoyment in any sports activities because enjoyment is closely related to adherence [24]. All these suggestions could have a practical impact on improving the fitness, self-esteem, confidence, and quality of life of children and adolescents with CP.

Our data and those of the literature suggest that it is important to consider involvement in sports activities for CP children; therefore, children with CP should be encouraged to continue with sports activities, even if for a short time only. Further studies performed in large populations and with different types of CP with a longer follow-up could be useful to confirm these results.

## Figures and Tables

**Table 1:** Details of the manuscripts included in the present review.

Study	Design	Population	Methods	Activity	Results after the Activity
Effects of creative dance-based exercise on gait performance in adolescents with cerebral palsy [20]	Single-group cohort study	10 adolescents (13–20 years), Spastic CP, GMFCS Level I–II	ROM GMFM-88 (D, E) BCS Spatio–temporal gait parameters Lower limb range	Creative dance classes, with 2-h sessions twice a week for 12 weeks	Significant improvements (*p* < 0.01) in GMFM-88 dimensions D-E, walking speed, cadence, step, stride length, sagittal ranges of motions of hip, and ankle ROM during walking. Significant decrease (*p* < 0.01) in the time of opposite foot off and first double-limb support time, significant increase (*p* < 0.01) in the percentage of single-limb support time. The BCS score increased after the intervention.
Dance program for physical rehabilitation and participation in children with cerebral palsy [21]	Pilot study	16 children (no age range reported) Spastic CP, (GMFCS I–IV)	Questionnaires	Dance classes once a week for 4–8 weeks	Significant improvement (*p* < 0.01) in enjoyment level and desire for more classes; parents reported significant (*p* < 0.01) perceived therapeutic benefit. Therapists viewed the class as a positive adjunct to therapy
Pilot study of a targeted dance class for physical rehabilitation in children with cerebral palsy [22]	Pilot study	12 children (7–15 years), Spastic CP, GMFC Level II–IV, divided into dance class group (*n* = 6) and control group (*n* = 6)	PBS QUEST	Dance class of 1 h, 3 times a week in a 4-week period	Significant improvements (*p* < 0.01) in the PBS in the targeted dance class group (before versus after and before versus 1-month follow-up comparisons)
Benefits of a Dance Intervention on Balance in Adolescents with Cerebral Palsy [23]	Pre–post design study with a double baseline	10 children (10–17 years), Spastic CP (diplegia, hemiplegia, quadriplegia) GMFCS Level I–III	PBS PRT	Dance session of 60 min, twice a week for 10 weeks	Significant improvement (*p* < 0.01) in the PBS total score and rhythm production
Benefits and Enjoyment of a Swimming intervention for Youth with Cerebral Palsy: an RCT study [24]	Randomized controlled design with single-blinding	14 children (7–17 years), Spastic CP (6 diplegia, 5 hemiplegia, 2 dyskinetic, 1 nonclassifiable), GMFCS I–III		Swimming session for 40–50 min, twice, for 20 weeks.	Significant improvement (*p* < 0.01) in walking and swimming skills and level of enjoyment
The effect of adapted sports in quality of life and biopsychosocial profile of children and adolescents with cerebral palsy [25]	Prospective study	17 children (range 6–18 years), Spastic CP (hemiplegia, diplegia) GMFCS I–II	IARRP PODCI CBCL	Swimming and side soccer from 1 to 3 times a week for 1 year	Significant improvement (*p* < 0.01) in the quality of life in the dimensions of transfer and mobility, upper extremity function, global function of IARRP, in the biopsychosocial profile (attention disorder syndrome, attention hyperactivity deficit disorder at CBCL). Children with diplegia obtained greater benefits (*p* < 0.01) than those with hemiplegia in relation to comfort and pain.
Effects of Participation in Sports Programs on Walking Ability and Endurance Over Time in Children With Cerebral Palsy [26]	Retrospective cohort study	97 children (6–20 years), Spastic CP, GMFCS I–III	TUG-modified 6-min walk 25-foot walk/run	Intensive sports summer session (swimming, tennis, dance, martial arts, basketball, soccer, baseball, and adaptive cycling) of 6 h/d, 5 d/wk for up to 4 weeks, for 9 years	Significant improvements (*p* < 0.01) in the TUG, modified 6-minute walk distance, and 25-foot walk/run over time, mainly in GMFCS level III (primarily use a hand-held mobility device to walk)
Alternative exercise methods for children with cerebral palsy. Effects of virtual vs. traditional golf training [27]	Samples sizes	19 children (6–12 years), Spastic hemiplegia, GMFCS I or II, received virtual (*n* = 9) or traditional (*n* = 10) golf training	GMFM-88, MAS SMWT, LST 6MWT, Curl Up Sit and Reach Modified, Thomas Balance tests	Virtual and traditional golf. Session of 60 min of warm up, golf training, and cool down, 3 days/week for 12 weeks	Both training methods lower extremity flexibility and muscle strength, aerobic endurance, and Gross Motor Function Measure-88. Virtual golf training group reported significant (*p* < 0.05) improvement in balance and lateral step-up tests than traditional golf
Game-Based Dual-Task Exercise Program for Children with Cerebral Palsy: Blending Balance, Visuomotor, and Cognitive Training: Feasibility Randomized Control Trial [28]	Single-blind, randomized controlled trial	20 children (4–10 years), CP GMFCS I–III, dived into: training group (CG) (*n* = 10) and experimental group (XG) (*n* = 10)	PBS, GMFM-88 MCTSIB, dual-task (DT) balance assessment	Computer Game-based dual-task balance, 3 sessions per week of 45 min for 12 weeks	Significant (*p* < 0.05) improvement in PBS, GMFM, and DT balance measures in the experimental group
Effects of a Gaming Platform on Balance Training for Children With Cerebral Palsy [29]	Randomized controlled trial	40 children (5–10 years) with CP (hemiparesis), GMFCS II–III–IV, divided into a control group (*n* = 20) using pc mouse and experimental group (*n* = 20) playing personal computer games using the platform	CoP sway BBS FAB TUG	Gaming platform session of 50 min, 5 times a week, for 12 weeks	The intervention group had significantly (*p* < 0.05) better performance in the CoP sway, sway velocity, and balance tests (BBS test and TUG test) than the control group.
Preliminary Study of the Effect of Training With a Gaming Balance Board on Balance Control in Children With Cerebral Palsy [30]	Randomized controlled trial	56 children (6–18 years) with CP, GMFCS I–III, divided into a control group (using hands with mouse) and experimental group (using foot with a new balance board)	PBS 2 min walking test	Nintendo session of 45 min, 3 times/week, for 12 weeks.	Significant (*p* < 0.01) reduction in postural sway (sway path; sway area), and a significant improvement (*p* < 0.01) in a performance of balance test in experimental group
Effects of Nintendo Wii-Fit^®^ video games on balance in children with mild cerebral palsy [31]	Randomized controlled trial	30 children (range 5–18 years) with CP (diplegic, hemiplegic, dyskinetic type), GMFCS I–III, divided into 2 groups. 1: NDT + conventional balance training; 2: NDT+ Wii balance games	TGGT Wee FIM	Nintendo Wii session for 50 min, 2 days/week for 12 weeks	Improvement in balance and independence in daily lives in both groups. Significant improvement (*p* < 0.01) in the Wii group in balance and Wee FIM
Does Nintendo Wii balance board improve standing balance? A randomized controlled trial in children with cerebral palsy [32]	Randomized controlled trial	32 patients (7–14 years) with CP, spastic hemiplegia, spastic diplegia GMFCS I–II, divided into two groups: Wii therapy and standard physiotherapy (*n* = 16) and intervention (*n* = 16)	CoP sway	Nintendo Wii balance board, 3 sessions per week over a period of 6 weeks and two follow-up assessments (4 additional weeks)	Significant (*p* < 0.01) reduction in the CoP sway in the intervention group. Post hoc analysis revealed that children with hemiplegia only benefited from Wii therapy
The effects of virtual reality-based bilateral arm training on hemiplegic children’s upper limb motor skills [33]	Single-subject experimental design	3 children with hemiplegia (no age range reported)	WMFT PMAL	Nintendo Wii games were played for 30 min in each of the 12 sessions.	Significant (*p* < 0.01) improvement in upper limb motor skills on the affected sides and in bilateral coordination ability after virtual reality-based bilateral arm training
Personalized balance games for children with cerebral palsy [34]	Pilot study	25 children (5–18 years) divided into experimental and control groups	TCMS DBT TUG COP-PL	8–9 game sessions for 15–20 min, totaling 150–160 min for 2 weeks of daily training	Significant (*p* < 0.05) improvement in TCMS scores and DBT results in the experimental group

## Abbreviations

BBS	Berg Balance Scale;
BCS	Body Cathexis Scale
CBCL	Child Behavior Checklist for ages 6–18
COP-PL	Center-of-Pressure Path Length
CoP sway	Center-of-pressure sway
CP	cerebral palsy
DBT	Dynamic Balance Test
IARRP	Instrument for the Evaluation of Results in Pediatrics Rehabilitation
ids	individuals
FAB	Fullerton Advanced Balance Scale
GMFM-88	Gross Motor Function Measure-88
LST	Lateral Step-up Test
MAS	Modified Ashworth Scale
MCTSIB	Modified Clinical Test of Sensory Integration in Balance
PMAL	Pediatric Motor Activity Log
PRT	Pediatric reach test
PODCI	Pediatric Outcome Data Collection Instrument
QUEST	Quality of Upper Extremity Skills Test
6MWT	Six-minute walk test
TCMS	Trunk Control Measurement Scale
TGGT	Timed Get Up and Go Test Sit
TUG	Timed Up and Go
Wee FIM	Wee-Functional Independence Measure
WMFT	Wolf Motor Function Test
